# Predictors of diastolic deceleration time of coronary flow velocity of infarct related and reference coronary artery assessed by transthoracic Doppler echocardiography in the chronic phase of successfully reperfused anterior myocardial infarction: relation to infarct size

**DOI:** 10.3389/fcvm.2023.1196206

**Published:** 2023-09-12

**Authors:** Vojislav Giga, Milorad Tesic, Branko Beleslin, Nikola Boskovic, Dragana Sobic-Saranovic, Ivana Jovanovic, Ivana Nedeljkovic, Ivana Paunovic, Srdjan Dedic, Ana Djordjevic-Dikic

**Affiliations:** ^1^ Cardiology Clinic, University Clinical Center of Serbia, Belgrade, Serbia; ^2^ School of Medicine, University of Belgrade, Belgrade, Serbia; ^3^ Insitute for Nuclear Medicine, University Clinical Center of Serbia, Belgrade, Serbia

**Keywords:** transthoracic Doppler echocardiography, diastolic deceleration time of coronary flow velocity, myocardial infarction, microvascular damage, infarct size, single photon emission tomography

## Abstract

**Introduction:**

High-frequency transthoracic Doppler echocardiography (TDE) enables the assessment of flow velocity and velocity pattern in different coronary arteries, including the assessment of diastolic deceleration time (DDT) of coronary flow velocity. Short DDT of infarct related artery (IRA) (<600 msec) in the acute phase of anterior myocardial infarction (MI) is the predictor of adverse left ventricular (LV) remodeling and prognosis. The significance of DDT of coronary flow velocity assessment in the chronic phase of anterior MI is not well established. Our study aimed to establish the predictors of DDT of the coronary flow velocity of infarct related (left anterior descendent-DDT of LAD) and reference coronary artery, evaluated by TDE, and to assess their relation to infarct size in the chronic phase of successfully reperfused first anterior MI.

**Methods:**

Our study included 40 consecutive patients (34 men, mean age 52 ± 12 years) one month after the first anterior STEMI and single vessel disease successfully treated with primary PCI. All patients underwent SPECT MPI for the assessment of LV volumes, ejection fraction, and percentage of the myocardium with fixed perfusion abnormalities and echocardiographic examination including the evaluation of DDT of IRA and reference coronary artery TDE.

**Results:**

DDT of LAD correlated significantly to the WMSI (*r* = −0.467, *p* = 0.002), LV end-systolic volume (*r* = −0.412, *p* = 0.008), LV ejection fraction (*r* = 0.427, *p* = 0.006), while the strongest correlation was observed between DDT of LAD and the extent of fixed perfusion abnormality (*r* = −0.627, *p* < 0.0001), Multivariate analysis revealed percentage of fixed perfusion abnormalities along with DDT of reference coronary artery as the independent predictors of DDT of IRA. DDT of IRA shorter than 886 msec predicts large fixed perfusion abnormalities (>20%) with a sensitivity of 89% and specificity of 62% (AUC 0.842).

**Conclusion:**

DDT of LAD assessed by TDE in the chronic phase of successfully reperfused first anterior MI is a usefull variable for the assessment of microcirculatory function that exclusively reflects the extent of microvascular damage and relates to infarct size.

## 1. Introduction

High-frequency transthoracic Doppler echocardiography enables the assessment of flow velocity and velocity pattern in different coronary arteries, including the assessment of diastolic deceleration time (DDT) of coronary flow velocity. The analysis of coronary blood flow pattern in infarct related artery (IRA) by transthoracic Doppler echocardiography in the acute phase of reperfused anterior myocardial infarction (MI) can reliably detect the presence of “no-reflow” phenomenon characterized by the steep deceleration in diastolic flow velocity, with DDT shorter than 600 msec, accompanied by the decrease in peak systolic velocity and systolic flow reversal (1). This pattern of coronary blood flow reflects the presence of microvascular obstruction with the consequent increase in microvascular resistance in the acute phase of MI due to cellular injury, local inflammatory processes, increased vascular permeability, interstitial edema, and increased extravascular resistance to blood flow (2, 3). The presence of steep DDT of coronary flow (less than 600 msec), assessed by transthoracic Doppler echocardiography or invasively using intracoronary flow wire, in the acute phase of MI is a marker of future adverse left ventricular (LV) remodeling and poor prognosis with higher mortality and hospitalization rates (4–8). In the convalescent phase, 7 days after MI, short DDT of coronary flow velocity in LAD correlates with the extent of microvascular obstruction (9) and infarct size (10) as assessed by cardiac magnetic resonance imaging (CMR).

It has been demonstrated that after acute ischemic injury, microvascular function gradually improves within one month with a subsequent increase of DDT of IRA (11, 12). Importantly this improvement is translated into the reduction of infarct size (13). However, there are conflicting data regarding the relation between DDT of IRA and infarct size in the chronic phase of MI (11, 14, 15).

During the acute phase of MI changes in coronary flow were observed not only in IRA but also in reference artery, with lower coronary flow velocity reserve and increased microvascular resistance (16, 17) that both improve over time. The potential relation between DDT of reference coronary artery and infarct size in the chronic phase of MI has not been analyzed previously.

Our study aimed to establish the predictors of DDT of the coronary flow velocity of infarct related and reference coronary artery, evaluated by transthoracic Doppler echocardiography, and to assess their relation to infarct size in the chronic phase of successfully reperfused first anterior myocardial infarction.

## 2. Materials and methods

### 2.1. Study population

Fifty consecutive patients with first anterior STEMI, successfully treated with primary percutaneous coronary intervention (PCI), defined as the absence of no-reflow and with the final TIMI flow grade 3, and single vessel disease were included in the study. Exclusion criteria were the presence of any but treated coronary lesion and atrial fibrillation (due to beat-to-beat variability of coronary flow velocity). Ten patients had poor acoustic windows and suboptimal Doppler signals during the evaluation of coronary flow (7 in the RCA region and 3 in the LAD region) and therefore, were excluded from further analysis — so the final number of studied patients was 40 [34 (85%) men, mean age 52 ± 12 years]. The study protocol was approved by our institution's medical ethical committee.

### 2.2. Study protocol

All patients underwent resting two-dimensional echocardiography with transthoracic color Doppler evaluation of coronary flow pattern of the left anterior descending artery (infarct related artery) and right coronary artery (reference artery), 30 ± 3 days after primary PCI. Transthoracic echocardiography was performed using a commercially available digital ultrasound system (Acuson Sequoia C256; Siemens Medical Solutions USA, Inc., Mountain View, CA) with a 3V2C multifrequency transducer using second-harmonic technology. All standard echocardiographic views were obtained. A 17-segment model was used to determine LV function (18). Segmental wall motion was graded as follows: 1 = normal, 2 = hypokinetic, 3 = akinetic, and 4 = dyskinetic. The wall motion score index (WMSI) was obtained by dividing the sum of individual visualized segment scores by the number of visualized segments. Severe impairment of LV contractility was defined as WMSI > 1.5 (19).

### 2.3. Transthoracic Doppler echocardiographic evaluation

Transthoracic Doppler echocardiography was performed using the same ultrasound unit. After standard examination, distal left anterior descending coronary artery and right coronary artery flow were evaluated using a 4-MHz transducer. In color Doppler flow mapping, the velocity range was set from 16 cm/s to 24 cm/s. For distal left anterior descending coronary artery examination, the acoustic window was around the midclavicular line in the fourth and fifth intercostal spaces in the left lateral decubital position. For posterior descending coronary artery examination, the left ventricle was imaged in a standard apical two-chamber view. From this position, the transducer was slightly rotated anticlockwise and tilted anteriorly, until coronary blood flow in the posterior interventricular groove was identified by color Doppler. A sample volume (3–5 mm wide) was positioned on the color signal of the distal arterial segment. The spectral Doppler of the coronary artery flow showed a characteristic biphasic flow pattern with a larger diastolic component and a small systolic one. All studies of stop frames and clips were digitally recorded and stored for offline analysis. At each time point, three optimal diastolic flow profiles were measured and the results were averaged. DDT was measured from the peak diastolic velocity to the point of intercept of the initial decay slope with baseline (20). Echocardiographic examination and coronary Doppler flow velocities analysis were performed by 2 experienced investigators. We previously reported an interobserver agreement for CFVR evaluation of 90% in our laboratory (21).

### 2.4. SPECT MPI

All patients underwent gated SPECT MPI the next day after the echocardiographic examination with the investigator unaware of the results of previous measurements. 740 MBq of 99mTc-MIBI was injected 10–15 min after sublingual administration of 0.5 mg nitroglycerin coinciding with the peak hemodynamic response, as previously reported (22). The acquisition was performed 45–60 min after the injection. Gated SPECT MPI data were acquired in the supine position with the single head SPECT gamma camera (Siemens, e.cam) equipped with a high-resolution low energy collimator. Sixty-four projection images over a 180° semicircular orbit extending from the 45° right anterior oblique position to the 135° left posterior oblique position were acquired. Time per projection was 20 s, matrix size 64 × 64, zoom 1.45, and gating 8 frames per cardiac cycle. Using the e.soft commercial software, transaxial tomograms were generated from gated projection data, reconstructed with a filtered back-projected algorithm, and reoriented to obtain oblique-angle tomograms parallel to the long and short axes of the left ventricle. The reconstructed data were projected as myocardial tomographic slices in short-axis, vertical-long, and horizontal-long axis views. The 4D-MSPECT software was then used for semiquantitative evaluation of myocardial perfusion and function. The extent of myocardial perfusion abnormalities (%) was expressed relative to the left ventricle, based on polar maps. The extent and severity of perfusion abnormalities were evaluated by segmental analysis of MIBI uptake using the 17-segment model with the five-point scoring system according to the European Association of Nuclear Medicine/European Society of Cardiology guidelines for radionuclide imaging of cardiac function (23). The total score of MIBI uptake (SRS) was calculated. The conversion of SRS to the percentage of myocardium fixed perfusion abnormalities was accomplished by dividing the SRS by 68 (the worst segmental score possible) and multiplying by 100 (24). Large myocardium fixed perfusion abnormality was defined as fixed perfusion abnormality ≥20% (4). We also evaluated LV ejection fraction, end-diastolic volume (EDV), and end-systolic volume (ESV) using an automated algorithm for the determination of left ventricle surfaces from gated perfusion SPECT (24). Impaired LV systolic function was defined as LV ejection fraction <50%. The 95% limits of agreement for interobserver and intraobserver variability for EF were 0.2% ± 5.9% and 0.4% ± 3.5%, for SRS 0.5% ± 4.2% and 0.4% ± 4.4%, and for the extent of perfusion abnormalities 0.5% ± 4.1% and 0.2% ± 3.3%, respectively (25).

### 2.5. Statistical analysis

The continuous data are expressed as a mean ± standard deviation. Normal distribution of all data was confirmed by Kolmogorov–Smirnov test. Univariate analysis was used to evaluate the relationship between various echocardiographic variables and SPECT, heart rate, and DDT of infarct related and reference coronary artery. To select covariates independently associated with DDT and fixed perfusion abnormality, significant univariate predictors were reassessed by multivariate logistic analysis, with values for inclusion and elimination set at *p* < .05. Pearson's correlation was used to evaluate the relation between various echocardiographic, nuclear and clinical data. Intergroup differences for continuous variables were tested using a 2-sided *T* test. To determine the best cut-off value of DDT for predicting large fixed perfusion abnormality maximal specificity and best sensitivity were determined based on ROC analysis. Sensitivity and specificity were calculated in the standard manner. *p-*values <.05 were considered statistically significant.

## 3. Results

**Figure 1:**
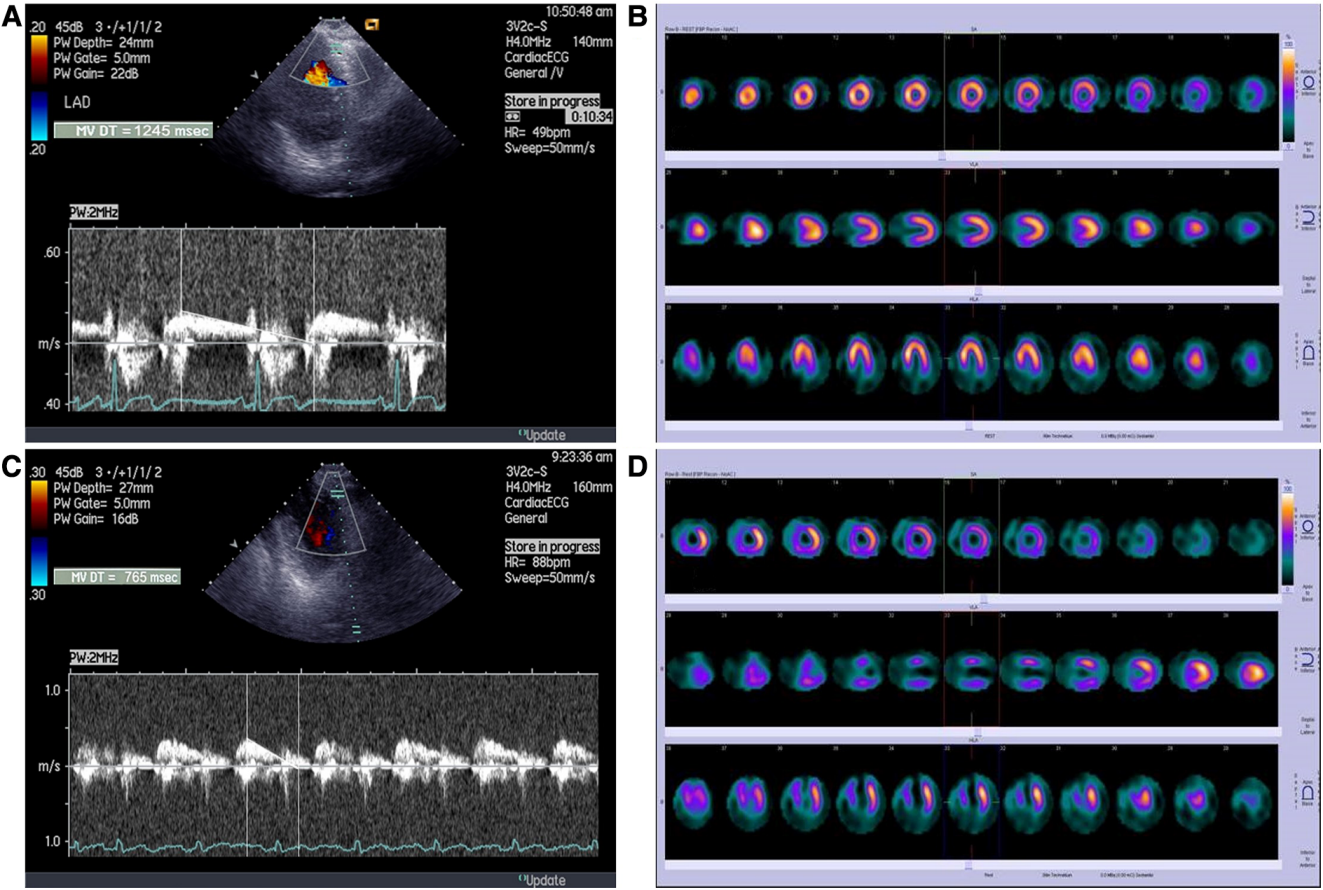
Representative tracings of (A) long DDT of LAD coronary flow velocity B) and SPECT MPI in patient without perfusion abnormalities; (C) short DDT of LAD coronary flow velocity and (D) SPECT MPI in a patient with large fixed perfusion abnormality of 43%.

Baseline characteristics of study population are summerized in Table 1. All the patients were on dual antiplatelet therapy (aspirin and clopidogrel) and on beta blockers. Thirty-seven patients (93%) were on statin therapy, while ACE inhibitors received 32 (80%) patients. Peak serum creatine kinase activity was 3,089 ± 2,347 IU (range 229–8,984 IU). Overall, one month after primary PCI, SPECT MPI revealed well preserved LV volumes and systolic function with a wide range of values. The percent of LV exhibiting fixed perfusion abnormalities on SPECT MPI was 24% ± 17% (range 0%–62%). Echocardiography revealed moderately impaired WMSI in our patient population, with patients without wall motion abnormalities on one side and those with severe contractile impairment on other side (Table 2). Representative tracing of DDT of LAD coronary flow velocity and SPECT MPI are presented in Figure 1.

**Table 1:** Baseline characteristics (*N* = 40 patients).

Age, years	52 ± 12
Male, *n* (%)	34 (85)
Diabetes, *n* (%)	4 (10)
Hypertension, *n* (%)	22 (55)
Dyslipidemia, *n* (%)	30 (75)
Smoking, *n* (%)	24 (60)
Peak CK (IU) activity	3,089 ± 2,347
Infarct location	
Ostial LAD, *n* (%)	2 (5)
Proximal LAD, *n* (%)	18 (45)
Mid LAD, *n* (%)	19 (47.5)
Distal LAD, *n* (%)	1 (2.5)
Number of stents	1.2 ± 0.5
Total stent length (mm)	23.4 ± 8.0
Stent diameter (mm)	3.3 ± 0.3

CK, creatine kinase; LAD, left anterior descending.

**Table 2:** SPECT MPI and echocardiographic data (*N* = 40 pts).

Variable	Mean ± SD	Range
End-diastolic volume (ml)	150 ± 65	62–338
End-systolic volume (ml)	79 ± 55	18–235
Ejection fraction (%)	52 ± 14	27–74
Fixed perfusion abnormality (%)	24 ± 17	0–62
WMSI^a^	1.56 ± 0.44	1.00–2.30
Peak diastolic flow velocity LAD (msec)	0.27 ± 0.06	0.18–0.40
Peak diastolic flow velocity RCA (msec)	0.26 ± 0.05	0.17–0.41
DDT^a^ of LAD coronary flow velocity (msec)	1,008 ± 225	321–1,507
DDT^a^ of RCA coronary flow velocity (msec)	1,093 ± 188	680–1,438

WMSI, wall motion score index; DDT, diastolic deceleration time; LAD, left anterior descendent; RCA, right coronary artery.

^a^ Assessed by echocardiography.

### 3.1. Transthoracic color Doppler evaluation of coronary flow velocity pattern in infarct related and reference coronary artery

TDE evaluation of the coronary flow of the left anterior descendent artery was feasible in 47 out of 50 patients (94%), whereas adequate visualization of right coronary artery flow was feasible in 43 out of 50 patients (86%), with final study population of 40 patients. The mean heart rate at the beginning of DDT of coronary flow velocity measurement was 70 ± 13 beats/min, systolic blood pressure was 126 ± 12 mmHg, whereas diastolic blood pressure was 74 ± 9 mmHg. There was no difference in peak diastolic flow velocity of IRA (LAD) and reference artery (RCA), *p* = 0.382. However, the DDT of LAD coronary flow velocity was significantly shorter than the DDT of RCA coronary flow velocity, *p* = 0.017 (Table 1). DDT of LAD flow was not related to the peak diastolic velocity (*r* = −0.162, *p* = 0.317), but there was a significant correlation between DDT of LAD flow and heart rate (*r* = −0.628, *p* < 0.001). Also, there is a significant correlation between DDT of LAD flow and DDT of reference coronary artery flow (*r* = 0.494, *p* = 0.001).

**Figure 2:**
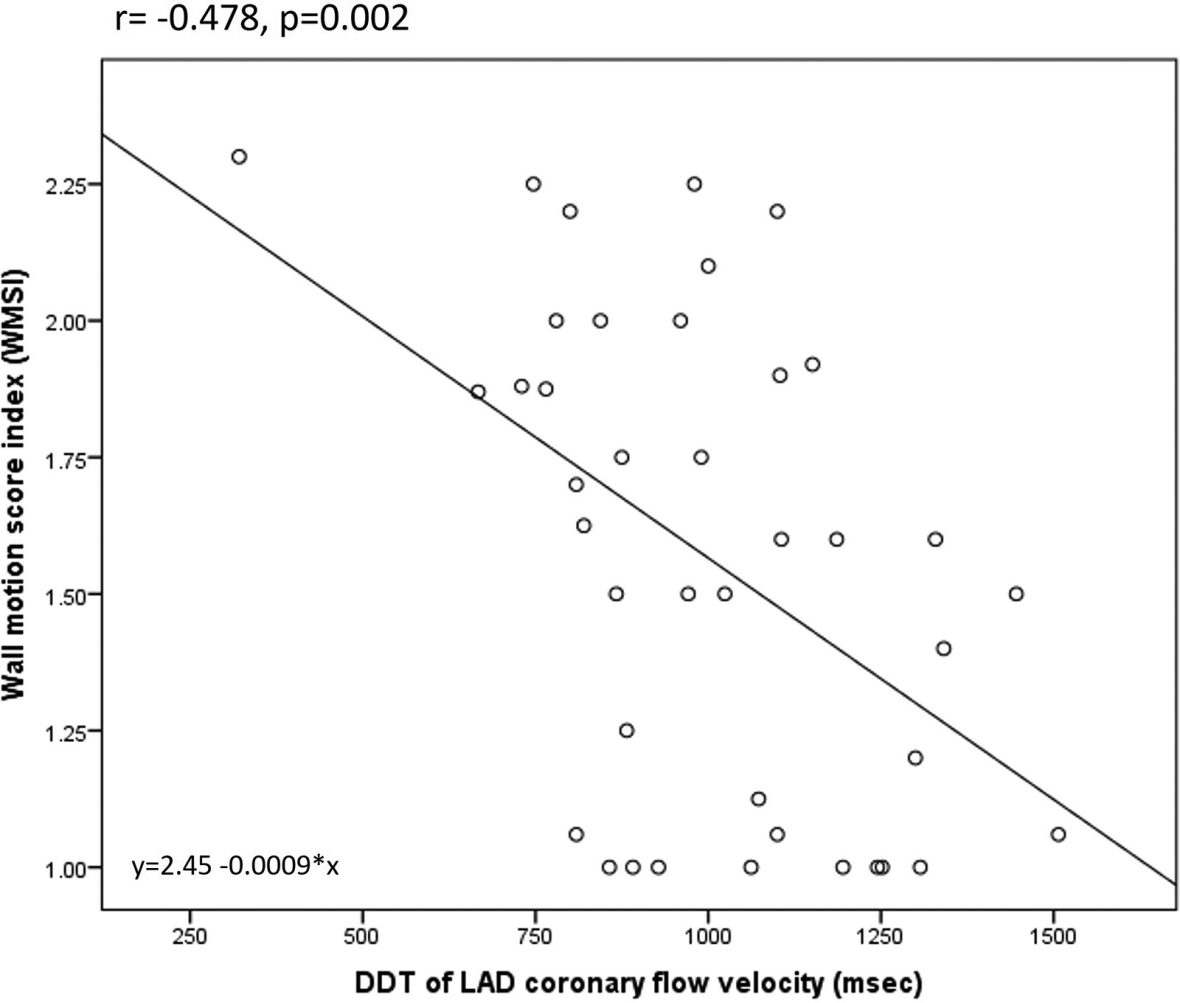
Correlation between DDT of LAD coronary flow velocity and wall motion score index.

**Figure 3:**
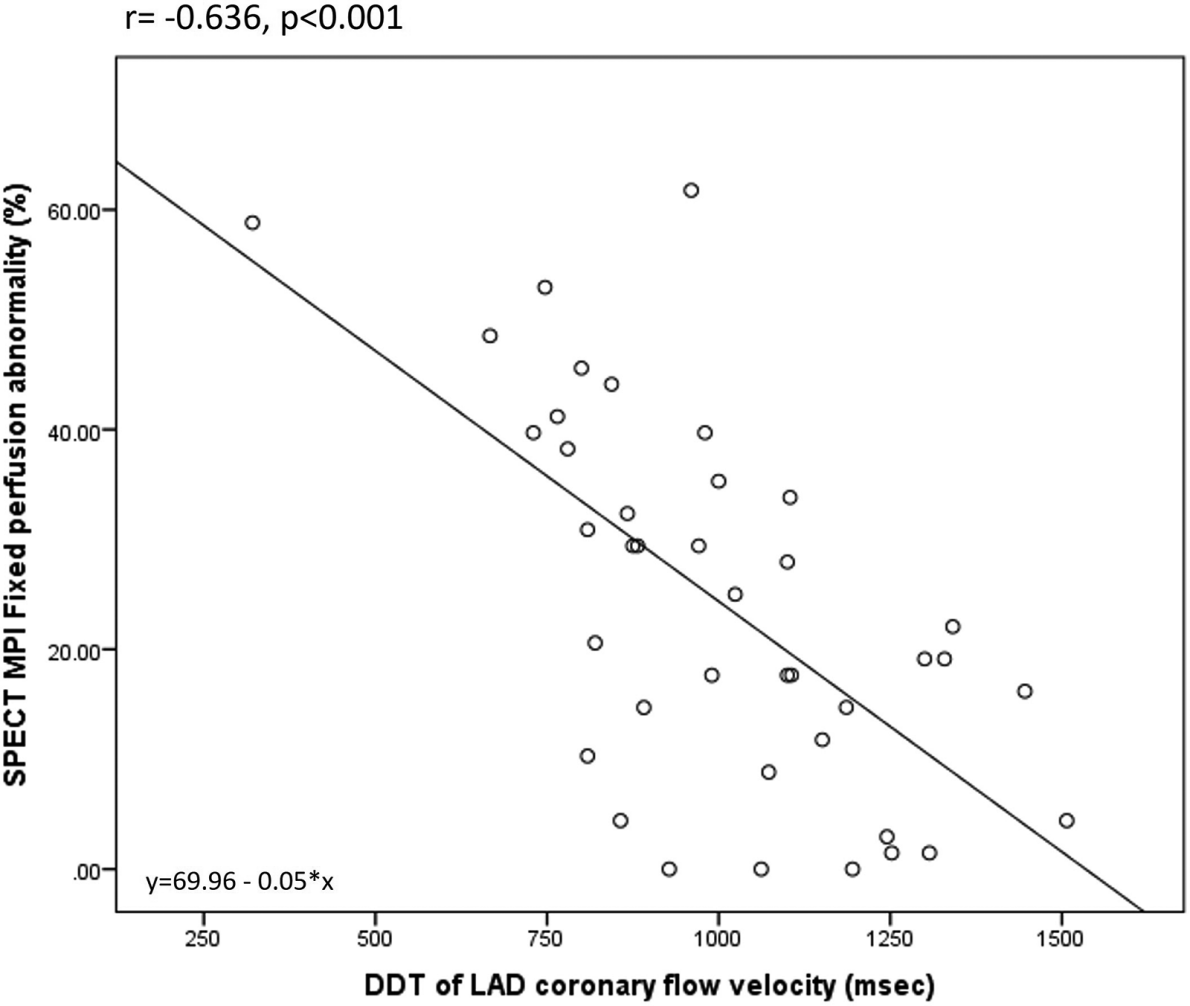
Correlation between DDT of LAD coronary flow velocity and SPECT MPI fixed perfusion abnormality.

Overall, DDT of LAD was significantly related to the different parameters of LV contractility, remodeling, and global systolic function. There was a significant negative correlation between DDT of LAD and WMSI assessed by echocardiography (*r* = −0.478, *p* = 0.002). Also, DDT of LAD correlated significantly to the LV end-systolic volume (*r* = −0.429, *p* = 0.006) and LV ejection fraction (*r* = 0.442, *p* = 0.004), while there was no statistically significant correlation with LV end-diastolic volume (*r* = −0.308, *p* = 0.053) assessed by SPECT MPI. The strongest correlation was observed between DDT of LAD and the extent of fixed perfusion abnormalities assessed by SPECT MPI (*r* = −0.636, *p* < 0.0001) (Figures 2,3).

Multivariate analysis revealed the percentage of fixed perfusion abnormalities and DDT of reference coronary artery as the independent predictors of DDT of LAD coronary flow velocity. Univariate and multivariate predictors of DDT of LAD are presented in Table 3.

**Table 3:** Univariate and multivariate predictors of DDT of LAD coronary flow velocity.

Variable	Univariate analysis			Multivariate analyisis		
	B coefficient	95% CI	*p*-value	B coefficient	95% CI	*p*-value
		Lower to upper			Lower to upper	
Age (years)	−3.67	−10.07–2.73	0.157			
End-diastolic volume (ml)	−1.13	−2.27–0.14	0.053			
End-systolic volume (ml)	−1.84	−3.10 to −0.57	0.006	0.32	−1.45–2.09	0.716
Ejection fraction (%)	7.33	2.45–12.21	0.004	−3.80	−12.20–4.61	0.365
Fixed perfusion abnormality (%)	−8.88	−12.41 to −5.34	<0.001	−11.15	−17.00 to −5.29	<0.001
WMSI^a^	−258.11	−414.06 to −102.17	0.002	36.69	−201.82–275.20	0.756
Heart rate (beats/min)	−10.63	−15.34 to −5.93	<0.001	−4.39	−8.90–0.12	0.056
Peak diastolic velocity (m/s)	−789.62	−2,062.21–482.98	0.217			
DDT of RCA coronary flow velocity (msec)	0.62	0.263–0.985	0.001	0.47	0.18–0.76	0.002

WMSI, wall motion score index; DDT, diastolic deceleration time; RCA, right coronary artery.

^a^ Assessed by echocardiography.

On the other hand, DDT of the reference coronary artery was not related to WMSI (*r* = −0,139, *p* = 0.813), LV end-diastolic (*r* = −0.61, *p* = 0.708), and end-systolic volume (*r* = −0.122, *p* = 0.455), LV ejection fraction (*r* = 0,135, *p* = 0.406 and the extent of fixed perfusion abnormality (*r* = −0,72, *p* = 0.658). DDT of the reference coronary artery was significantly related only to the heart rate (*r* = −0.815, *p* < 0.001).

Shorter DDT of LAD diastolic flow velocity was observed in patients with LV ejection fraction <50% (859 ± 217 ms vs. 1,080 ± 280 msec in those with LV ejection fraction ≥50%, *p* = 0.03), and in those with WMSI > 1.5 (908 ± 221 msec vs. 1,108 ± 213 msec in those with WMSI ≤ 1.5, *p* = 0.006). Patients with large fixed perfusion abnormalities >20% also had significantly shorter DDT of coronary flow velocity in LAD in comparison to the patients with smaller perfusion defects (875 ± 201 msec vs. 1,143 ± 194 msec, *p* < 0.001). By multivariate analysis DDT of LAD flow and WMSI assessed by echocardiography were independent predictors of large fixed perfusion abnormalities. Univariate and multivariate predictors of fixed perfusion abnormality >20% are presented in Table 4.

**Table 4:** Univariate and multivariate predictors of fixed perfusion abnormality.

Variable	Univariate analysis			Multivariate analyisis		
	B coefficient	95% CI	*p*-value	B coefficient	95% CI	*p*-value
		Lower to upper			Lower to upper	
Peak CK	0.005	0.003–0.007	<0.001	0.64	−0.02–0.02	0.949
End-diastolic volume (ml)	0.174	0.110–0.238	<0.001	0.26	−0.209–0.261	0.823
End-systolic volume (ml)	0.230	0.163–0.296	<0.001	0.32	−0.355–0.399	0.861
Ejection fraction (%)	−0.947	−1.182 to −0.712	<0.001	−0.163	−0.829–0.502	0.619
WMSI^a^	32.768	25.988–39.548	<0.001	18.099	6.425–29.772	0.004
Heart rate (beats/min)	0.553	0.174–0.932	0.005	−0.163	−0.422–0.122	0.255
Peak diastolic velocity (m/s)	98.808	11,693–186.124	0.027	35.924	−28.059–98.648	0.264
DDT of LAD coronary flow velocity (msec)	−0.046	−0.064 to −0.027	<0.001	−0.25	−0.41–0.008	0.005

CK, creatin kinase; WMSI, wall motion score index; DDT, diastolic deceleration time; LAD, left anterior descending.

^a^ Assessed by echocardiography.

**Figure 4:**
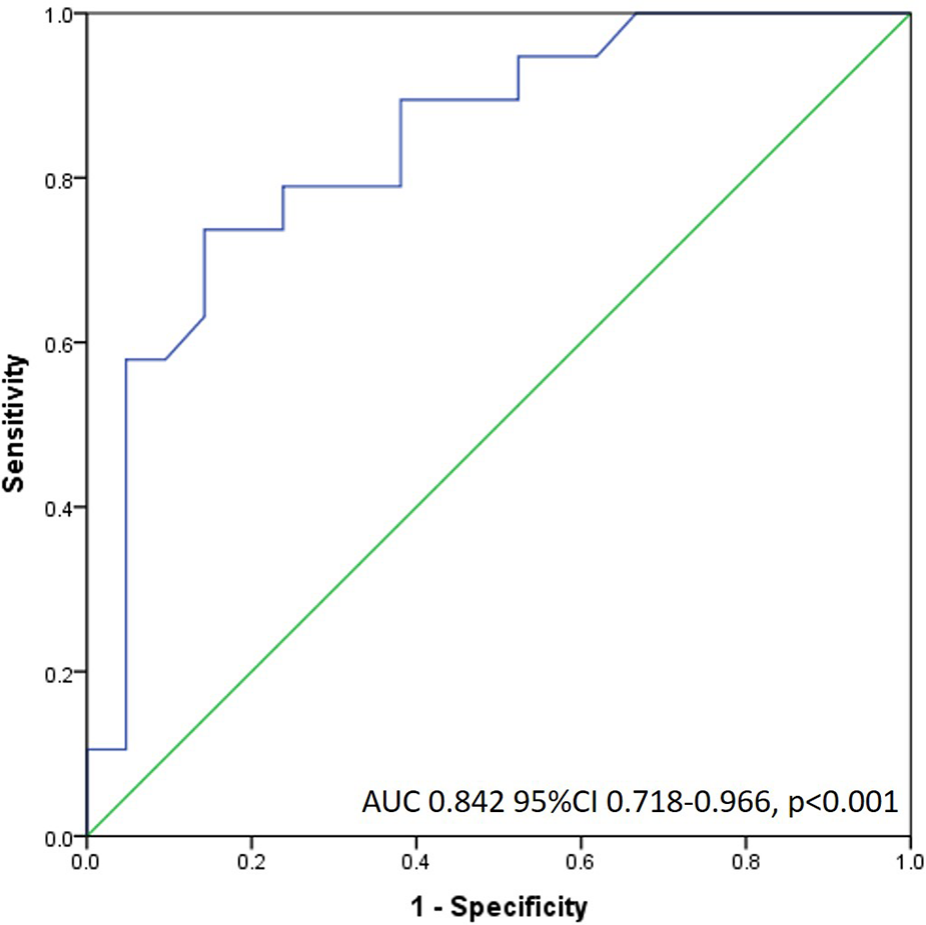
Receiver operating characteristic curve of DDT LAD flow for the detection of large fixed perfusion abnormality.

Specifically, by ROC analysis, DDT of less than 886 msec can identify large perfusion abnormalities with a sensitivity of 89%, and specificity of 62% (AUC 0.842) (Figure 4). The same cut-off value of DDT of LAD of less than 886 msec can identify patients with significant contractile impairment (WMSI > 1.5) with acceptable sensitivity of 79% and specificity of 56% (AUC 0.742) and impaired LV global systolic function (LV ejection fraction <50%) with sensitivity of 73% and specificity of 58% (AUC 0.757). The value of DDT of LAD of more than 1,102 msec virtually excludes all the patients with fixed perfusion abnormalities >20% (Specificity 99%). Adjustment of DDT of LAD coronary flow velocity for heart rate (by dividing DDT of LAD by heart rate) did not increase diagnostic capability for the identification of patients with fixed perfusion abnormalities >20% (AUC 0.815).

## 4. Discussion

In our study, we have demonstrated that the analysis of DDT of the coronary flow velocity of IRA (LAD), by transthoracic Doppler echocardiography, in the chronic phase of successfully reperfused first anterior myocardial infarction is a usefull variable for the assessment of microcirculatory function that exclusively reflects the extent of microvascular damage and is related to different variables depicting infarct size.

DDT of coronary flow velocity can be assessed either by intracoronary flow wire (1, 26) or by transthoracic Doppler examination (11, 20, 27). Non-invasive assessment with transthoracic Doppler echocardiography is highly feasible, with reported feasibility of 95% for all three major coronary arteries (100% for LAD, 90% for left circumflex artery, and 93% for RCA) as recently reported (10) with somewhat lower feasibility observed in our study (94% for LAD and 86% for RCA).

Functional measurement of microvascular injury, in the acute phase of MI, by intracoronary flow wire correlates well with anatomic microcirculatory damage assessed by gadolinium-enhanced magnetic resonance imaging (9) showing that the DDT value depends on the degree of microvascular damage and microvascular resistance (28). The higher extent of microvascular obstruction was associated with shorter DDT (*r* = −0.61, *p* = .001) (9). The majority of recovery from myocardial stunning occurs within 2 weeks after acute MI (29). However, this pattern of the steep decline of coronary flow in early diastole may persist in the chronic phase of anterior MI in patients with anteroapical myocardial scar, defined as the myocardial wall thickness <5 mm (15). Patients with definite scar have significant impairment of microcirculation with a smaller overall cross-sectional area and subsequently higher peripheral resistance (15). The remaining capillaries have decreased capacitance with rapid filling and fast abolition of the driving pressure gradient and shortened DDT of the LAD coronary flow velocity (30, 31). On the contrary, patients with preserved diastolic wall thickness (i.e., with less pronounced impairment of microcirculation) have coronary flow velocity pattern similar to the patients without previous acute ischemic event with a longer DDT of IRA (15). Analysis of coronary flow velocity pattern in patients one month after reperfused anterior MI in our study further confirms this pathophysiological concept. In the heterogenous population of patients 30 days after the first successfully reperfused anterior MI, DDT time of coronary flow velocity of IRA showed a wide range of values (521–1,507 msec). Diastolic deceleration time of IRA (LAD), in univariate analysis correlated significantly with different parameters of LV remodeling, namely end-systolic LV volume, global systolic function (ejection fraction), and contractile impairment (WMSI). However, the strongest correlation was observed with the extent of fixed perfusion abnormalities assessed by SPECT MPI, which remained the independent predictor of DDT of the coronary flow velocity of IRA in multivariate analysis. This finding supports the concept that DDT of coronary flow velocity assessed by transthoracic Doppler echocardiography reflects exclusively the extent of microvascular damage since the intact microcirculation is a prerequisite for MIBI uptake (i.e., perfusion abnormalities on SPECT MPI represent the areas of destructed microcirculation) and is not affected by the LV remodeling, function and contractile state. Our findings suggest that short DDT of LAD flow occurs due to permanent microvascular damage and is not affected by global ventricular function or contractility. This finding is furthermore supported by the fact that DDT of coronary flow velocity in the reference artery was not related to any parameter of LV function and remodeling and was not related to the extent of perfusion abnormalities in the myocardium supplied by IRA. Our results suggest that the analysis of DDT of the coronary flow velocity of IRA by TDE is a useful tool for the assessment of microcirculatory function even in the chronic phase of MI.

After an acute ischemic injury in the later phase of the MI microvascular function recovers, which is manifested by prolongation of the diastolic deceleration time in the IRA (6, 11, 12). This recovery of microcirculation is a consequence of the gradual recanalization of occluded small blood vessels and neoangiogenesis in the infarcted zone with an increase in the capacitance of intramyocardial circulation (32). The value of DDT of LAD flow in our study was comparable to the values obtained by Shintani et al. (11) three weeks after reperfused anterior MI. In contrast to our results, there was no difference in DDT of coronary flow velocity in patients with and without LV remodeling three weeks after MI (11). However, it should be noticed that DDT of the coronary flow velocity of IRA was significantly shorter (887 ± 442 msec) in patients with LV remodeling than in those with preserved (1,041 ± 197 msec) or modestly impaired (1,172 ± 288 mesc) LV functions and volumes assessed 6 months after an acute ischemic injury, although not reaching statistical significance. Similarly, Hozumi et al. (6) did not find a significant difference in DDT at 2 weeks after intervention between viable and nonviable myocardium, although DDT was longer in patients with viable myocardium (991 ± 75 vs. 795 ± 281 msec). However, there was a significant correlation between the DDT and the WMSI 14 days after the MI. Also, DDT measured by transthoracic Doppler echocardiography 4 weeks after MI remained significantly related to WMSI 6 months after acute injury (6). A study by Karatasakis et al. (15) including patients in the chronic phase of MI also confirmed the correlation between the extent of myocardial damage and pressure half-time of LAD diastolic flow velocity. In our study, we have demonstrated significantly shorter DDT of LAD diastolic flow velocity in patients with impaired global LV function (LV ejection fraction <50%) and in those with significantly impaired LV contractility (global WMSI > 1.5) thirty days after reperfused anterior MI, suggesting that DDT remains related to different prognostically important parameters of infarct size (33), even in its chronic phase. It should be noticed that global WMSI > 1.5, corresponds to LV ejection fraction <50%, as demonstrated by cardiac magnetic resonance imaging (34), and is comparable to regional WMSI > 2.0 in LAD territory that is used in previous studies assessing the relation between DDT of LAD flow and LV contractility and remodeling (6, 11). Importantly this relation is observed in the whole spectrum of patients that are seen in everyday clinical practice i.e., those with completely preserved (9 out of 40 patients in our study had WMSI = 1) to severely impaired LV function and contractility (7 out of 40 patients in our study had severe contractile impairment with global WMSI > 2.0). Similarly, patients with large fixed perfusion abnormalities >20% had also significantly shorter DDT of coronary flow velocity in LAD in comparison to the patients with smaller perfusion defects. DDT of LAD of less than 886 msec can predict severe damage of microcirculation, as assessed by SPECT MPI one month after MI with excellent sensitivity of 89% and moderate specificity of 62%. Diagnostic capability was not improved after the adjustment of DDT of LAD coronary flow for heart rate. Such a moderate specificity is mainly driven by the wide range of DDT of LAD in patients without or with small perfusion defects and may be partially explained by the huge variations of basal myocardial blood flow that are observed in healthy subjects (35). Furthermore, this concept is supported by our finding of the wide range of DDT in reference artery, i.e., in the artery with preserved integrity of microcirculation. Moreover, DDT of the reference coronary artery was an independent predictor of DDT of LAD suggesting that observed variation in DDT values is a biological phenomenon that is related to factors that alter properties of microcirculation as a whole. Despite the observed variability of DDT, its value in IRA higher than 1,102 msec virtually excludes all the patients with large perfusion abnormalities, whereas the value shorter than 886 msec identifies a high proportion of patients with severely damaged microcirculation. It is of importance that in our study the severity of fixed perfusion abnormality was independently related to DDT of LAD coronary flow. Imamura et al. demonstrated that DDT assessed in all three major infarct related coronary arteries is independently related to the presence of transmural myocardial infarction (TEI grade 4) as assessed by cardiac magnetic resonance imaging 7 days after acute MI (10). In addition to this result, our findings suggest that DDT of coronary flow remains related to the infarct size assessed by different imaging modality (SPECT MPI) also in the chronic phase of MI. In aforementioned study, the DDT cut-off value for the detection of TEI grade 4 (transmural MI) in three major vessels was determined as 950 msec by the ROC curve (10). The lower cut-off value for DDT of coronary flow velocity of 886 msec in LAD observed in our study can be partially explained by the fact that we included only patients with larger anterior MIs. In the study by Hirsch et al., which included exclusively patients with anterior MIs, mean DDT of LAD coronary flow assessed by Doppler wire during recatheterization within 8 days after primary PCI was 708 ± 262 msec in patients without microvascular obstruction on cardiac magnetic resonance imaging, whereas in patients with severe microvascular obstruction mean DDT of LAD coronary flow was 382 ± 142 msec (9). Additionally, observed difference in cut-off values can be explained by the use of more sophisticated technique (cardiac magnetic resonance imaging) for the infarct size estimation in the study by Imamura et al.

In the acute phase of MI coronary flow is impaired both in infarct related and reference coronary artery, with gradual improvement over time (16, 17). However, myocardial blood flow remained lower both in infarct related and reference coronary artery in comparison to healthy individuals (16). It seems that microvascular function does not recover to the same extent in IRA and reference coronary artery, as demonstrated by the shorter duration of DDT of LAD than of reference artery in the current study. These differences between DDT of LAD and reference coronary artery could be used for the explanation of which part of the decrease in DDT was caused by microvascular dysfunction as a global phenomenon of coronary circulation, caused by traditional risk factors, compared to a decrease in DDT caused together by microvascular dysfunction and obstruction with loss of capillaries due to tissue scarring. The current finding of shorter DDT of IRA than of reference artery is concordant with our previous finding of lower coronary flow velocity reserve of LAD in comparison to coronary flow velocity reserve of RCA-PD one month after successfully reperfused anterior MI (36).

Previously, we have demonstrated, in the subset of current study population, that coronary flow reserve derived percentage of microvascular damage (CFR-PMD), parameter calculated from coronary flow velocity reserve of infarct related and reference coronary artery (assessed after hyperemic stimulus) is related to various indices of myocardial infarct size in its chronic phase (36). Present study provide an evidence that the duration of DDT of coronary flow velocity of IRA, a simple parameter that is assessed in basal condition, without a need for the induction of hyperemic response, is related to the LV function, contractility and can reliably identify patients with extensive fixed perfusion abnormality on SPECT MPI in the chronic phase of the first anterior MI.

### 4.1. Study limitations

Our study sample was small and consisted only of patients with previous anterior MI and single vessel disease successfully treated with primary PCI with final TIMI flow grade 3, so our data cannot be extrapolated to the patients with inferior or lateral MI, and to those with lower TIMI grade. In-stent restenosis cannot be completely excluded since the patients didn't have an angiographic follow-up one month after the revascularization. However, it has been demonstrated that the analysis of DDT of the coronary flow velocity of IRA before revascularization can identify those patients with previous MI who have the potential of functional recovery after elective percutaneous coronary intervention (21), suggesting that the evaluation of DDT of IRA can provide insight into microcirculatory function even in the presence of coronary artery stenosis. Finally, we did not use contrast agents to enhance Doppler signals and improve the feasibility of coronary flow velocity assessment in the infarct related and reference coronary artery.

## 5. Conclusion

The analysis of DDT of the coronary flow of IRA, by transthoracic Doppler echocardiography, is a simple, easily obtainable, and highly feasible parameter that provides insight into the extent of microvascular damage in the chronic phase of successfully reperfused anterior myocardial infarction. A shorter duration of DDT is related to adverse LV remodeling, impaired global systolic function and contractility. Specifically, the shorter duration of DDT flow of the LAD artery is related to the higher extent of fixed perfusion abnormalities as assessed by SPECT MPI. DDT of coronary flow velocity in the reference artery is not related to the infarct size in the chronic phase of MI providing further evidence that DDT assessed by transthoracic Doppler echocardiography in IRA reflects the extent of microvascular injury.

## Data Availability

The raw data supporting the conclusions of this article will be made available by the authors, without undue reservation.
